# Supplementary material for: Gastroesophageal reflux disease in Sri Lanka: An island-wide epidemiological survey assessing the prevalence and associated factors
Source: PLOS Glob Public Health. 2024 May 1;4(5):e0003162. doi: 10.1371/journal.pgph.0003162 (PMC11062562; doi:10.1371/journal.pgph.0003162)
Supplement: Attachment — Submitted filename: Dear Authors.docx [file pgph.0003162.s002.docx]

**Dear Authors:**

**Manuscript Title**: Gastroesophageal Reflux Disease in Sri Lanka: an island-wide epidemiological survey assessing the prevalence and associated factors

I have read your manuscript. As you have mentioned the prevalence of GERD is rising all over the world, and not only in the western world. As the first country-wide epidemiologic study is important and will be read and cited is global epidemiological studies and as you are filling the missing data is of importance.

As a general and in detail view of the manuscript there are some points to say:

- The manuscript is very long and there are many repeated items in the text that could be omitted
- The main structure is like a research report rather that a medical journal manuscript. You should not tell all of the findings you had found in the study, which were obvious and adds nothing to global knowledge. I think it is better to focus on your best and the most important findings and the items that are different from other parts of the world or alike the other parts that are important to mention.
- I think you have used the term of “reflux” instead of “(acid) regurgitation” interchangeably in the text. The symptom regurgitation differs from the reflux process and reflux disease!
- In the sample enrollment process, there is nothing about how you came to the number of 1200 people and how you made your clusters from the country. You had lost some 50 persons due to some reasons that is obvious, but I do not think that the method you enrolled new people is methodologically correct.
- The term “bloating” as a symptom of GERD disease seems not proper. You may have considered “belching”. If otherwise please give some reference to this symptom in GERD patients.
- Many items in the tables and main text can be shortened to include only the necessary points and facts to be shown.
- In the discussion section there is a section, we it is said that NSAIDs can induce GERD. They are a well-known etiology for upper GI erosions/ ulcers and also dyspeptic symptoms, but not a main factor in the GERD.
- For the figure 1, I think the box plot type may be a better format concerning the age group chosen in the chart

**Best Regards**
