# Supplementary material for: Gastroesophageal reflux disease in Sri Lanka: An island-wide epidemiological survey assessing the prevalence and associated factors
Source: PLOS Glob Public Health. 2024 May 1;4(5):e0003162. doi: 10.1371/journal.pgph.0003162 (PMC11062562; doi:10.1371/journal.pgph.0003162)
Supplement: Attachment — Submitted filename: Response to Reviewers.docx [file pgph.0003162.s004.docx]

15^th^ February 15, 2024

Dr. Nilanka Wickramasinghe

Department of Physiology

Faculty of Medicine

University of Colombo

Editor

PLOS Global Health

Dear Sir/Madam,

I am pleased to send our manuscript titled “Gastro-esophageal Reflux Disease in Sri Lanka: an island-wide epidemiological survey assessing the prevalence and associated factors” following the revisions made under the advice of the second reviewer. I hope that my manuscript will be accepted.

Do let me know if there is anything to be corrected.

I’m pinning much hope on this research as it is the main study in my PhD thesis.

Given below is the response to reviewer's comments.

Thanking you

Sincerely

Nilanka Wickramasinghe

Review Comments to the AuthorReviewer #2: Wickramasinghe and colleagues report on an epidemiological survey assessing the prevalence of GERD and associated factors in Sri Lanka. There is limited data from that part of the world so understanding GERD locally is a compelling reason to perform the investigation. The methods are appropriate for the stated aim of the study. However, there are some issues in the paper in its present form that preclude publication with the present version.

Major issues:

- Results line 181: For clarity that sentence should mention probable weekly GERD was 25.3%. Do you have weekly heartburn, regurgitation and heartburn + regurgitation prevalence values? That should be included. Was daily overall prevalence along with breakdown above obtained as well, if so that should be included in section as well.

Heartburn and or regurgitation at once weekly was considered probable GERD according to the worldwide definitions used in systematic reviews on GERD. Table 1 describes the questions asked. We will also include it in the paragraph as suggested by the reviewer for more clarity. Line 181 is rewritten as given below.

“In this study, the prevalence of probable GERD (heartburn and or regurgitation weekly) was 25.3% (304). The prevalence of subjects with heartburn weekly and daily is 56.6% and 25.3% while for regurgitation it is 42.2% and 15.8% respectively. Age and sex-specific prevalence of probable GERD is shown in **Fig 1**. “

- Results line 230-231: antihypertensive medications were independently associated with GERD. Was a sub analysis of antihypertensive types taken done to determine if this finding was associated with a particular drug class (calcium channel blocker, diuretic, beta blocker, ACE or ARB). Believe that is important to know.

A sub analysis of antihypertensives were not done as we questioned the participants only the use of antihypertensives and not the particulars or types of antihypertensive medications used. This was due to issues related to identifying the correct medication when dealing with laymen. Furthermore, in Sri Lanka, prescriptions are often not available with the patients. This is clarified by adding another line to text at 231 line as given below.

“Certain medications used such as statins [adjusted OR = 1.740, p = 0.021] and antihypertensive medications [adjusted OR =1.508, p = 0.060] were independently associated with GERD. The subtypes of medication were not questioned from the participants.”

- Results table 4: In the probable GERD column/ type of medication row box, the total adds up to more than the number in the Probable GERD group suggesting that some patients use multiple medications. Is that number known and if so, it should be included. Also, the control column/ type of medication box, the total does not add up to the number of controls suggesting that some did not take any medication. That should also be reported.

Line 250 is rewritten to highlight the important point showed by the reviewer.

Of the 304 probable GERD subjects, 213 (70.1%) used some form of medication for GERD symptoms. This contrasts with the value of 27.7% which is the percentage of controls on medication for GERD symptoms. Of the 304 probable GERD subjects, 64 (21.1%) were on more than one medication for GERD symptom control.

- Discussion lines 315-316: This sentence needs to be changed as the comparison between daily heartburn and regurgitation rates in Turkey are compared to weekly in Sri Lanka. This is not a legitimate comparison. The appropriate one is either week to week or daily to daily.

We have rewritten the sentence as the reviewer has suggested to make it clearer.

When comparing the results of a similar study in Turkey to ours in Sri Lanka, the prevalence of daily symptoms of heartburn (1.2%) and regurgitation (1.2% ) in Turkey, was lower than the daily symptoms of heartburn and regurgitation reported in our study (25.3% and 15.8% respectively) [26].

- Some minor issues in the revision that should be corrected:
  Abstract lines 44-45: sleeping within two hours of consuming a meal is mentioned twice, the second one should be removed.

Thank you for pointing out the error. It will be removed.

- Introduction line 65: Barret’s should be Barrett’s.

Thank you for pointing out the spelling mistake. It will be corrected.

21/10/2023

Dear Editor

In response to the comments by the editors on 11/12/2023:

1. ethics extension.pdf, ethics pdf colombo.pdf and STROBE-checklist-v4.docx are currently uploaded as file type “Other”, which is not viewable by the reviewers. Please change the file type(s) to 'Supporting Information' and include a legend in the manuscript if you wish it/them to be included in review.

1. ethics extension.pdf, ethics pdf colombo.pdf and STROBE-checklist-v4.docx are documents containing my certification from the ethics review committee. I have added it as "other" for the editors to double-check as these documents were asked in other PLOS journals. Would you like for me to include them as supplementary files in the manuscript for the reviewers to see as well? If it is not required i don't mind taking it down. I do not think it is needed for the manuscript review process but for editors. Please advise.

2. Figure 2: Please confirm (a) that you are the photographer; or (b) provide written permission from the photographer to publish the photo(s) under our CC-BY 4.0 license.

Figure 2 is a color map diagram showing the prevalence of individual districts I have created (Nilanka Wickramasinghe) and have not copied anyone. The basic shape of Sri Lanka is from the Population Census layer maps- 2012- Department of Census and Statistics Sri Lanka website, which is open access. The majority of the figure is my creation.

 This is mentioned already in my revised manuscript where the source of the base layer is declared as "Census layer maps- 2012- Department of Census and Statistics Sri Lanka"

3. In the online submission form, you indicated that "The datasets used and/or analyzed during the current study are available from the corresponding author upon reasonable request". All PLOS journals now require all data underlying the findings described in their manuscript to be freely available to other researchers, either 1. In a public repository, 2. Within the manuscript itself, or 3. Uploaded as supplementary information.
This policy applies to all data except where public deposition would breach compliance with the protocol approved by your research ethics board. If your data cannot be made publicly available for ethical or legal reasons (e.g., public availability would compromise patient privacy), please explain your reasons by return email and your exemption request will be escalated to the editor for approval. Your exemption request will be handled independently and will not hold up the peer review process, but will need to be resolved should your manuscript be accepted for publication. One of the Editorial team will then be in touch if there are any issues.

3. The data is submitted to a public repository and this site and link are submitted in the response to reviewers' document as well as the data review URL in the online manuscript submission portal

 "The data has been submitted to Harvard dataverse. Link given below:

<https://doi.org/10.7910/DVN/D6AOWX>"

Sincerely

Nilanka

26^th^ September, 2023

Dear Editors and Reviewers,

Thank you very much for the comprehensive review of the paper. It helped to greatly enhance the research idea. We have done the required revisions. This document shows the point-by-point responses to the corrections and questions posed by both editors and reviewers. My responses are given in red text for ease of reading. Please let me know if there are any mistakes I can revise if not done so far.

Sincerely

Nilanka Wickramasinghe

Journal Requirements:

Comment

1. Please provide separate figure files in .tif or .eps format and remove them from the manuscript fle.

For more information about figure files please see our guidelines: 
<https://journals.plos.org/globalpublichealth/s/figures> 
<https://journals.plos.org/globalpublichealth/s/figures#loc-file-requirements>

Answer

Corrected accordingly.

Comment
2.  In the online submission form, you indicated that "The datasets used and/or analyzed during the current study are available from the corresponding author upon reasonable request.".

All PLOS journals now require all data underlying the findings described in their manuscript to be freely available to other researchers, either 1. In a public repository, 2. Within the manuscript itself, or 3. Uploaded as supplementary information.

This policy applies to all data except where public deposition would breach compliance with the protocol approved by your research ethics board. If your data cannot be made publicly available for ethical or legal reasons (e.g., public availability would compromise patient privacy), please explain your reasons by return email and your exemption request will be escalated to the editor for approval. Your exemption request will be handled independently and will not hold up the peer review process, but will need to be resolved should your manuscript be accepted for publication. One of the Editorial team will then be in touch if there are any issues.

Answer

The data has been submitted to Harvard dataverse. Link given below:

https://doi.org/10.7910/DVN/D6AOWX

Comment
3. Some material included in your submission may be copyrighted. According to PLOS’s copyright policy, authors who use figures or other material (e.g., graphics, clipart, maps) from another author or copyright holder must demonstrate or obtain permission to publish this material under the Creative Commons Attribution 4.0 International (CC BY 4.0) License used by PLOS journals. Please closely review the details of PLOS’s copyright requirements here: PLOS Licenses and Copyright. If you need to request permissions from a copyright holder, you may use PLOS's Copyright Content Permission form.

Please respond directly to this email or email the journal office and provide any known details concerning your material's license terms and permissions required for reuse, even if you have not yet obtained copyright permissions or are unsure of your material's copyright compatibility.

Potential Copyright Issues:

Figure 2: please (a) provide a direct link to the base layer of the map (i.e., the country or region border shape) and ensure this is also included in the figure legend; and (b) provide a link to the terms of use / license information for the base layer image or shapefile. We cannot publish proprietary or copyrighted maps (e.g. Google Maps, Mapquest) and the terms of use for your map base layer must be compatible with our CC-BY 4.0 license.

Note: if you created the map in a software program like R or ArcGIS, please locate and indicate the source of the basemap shapefile onto which data has been plotted.

If your map was obtained from a copyrighted source please amend the figure so that the base map used is from an openly available source. Alternatively, please provide explicit written permission from the copyright holder granting you the right to publish the material under our CC-BY 4.0 license.

Please note that the following CC BY licenses are compatible with PLOS license: CC BY 4.0, CC BY 2.0 and CC BY 3.0, meanwhile such licenses as CC BY-ND 3.0 and others are not compatible due to additional restrictions.

If you are unsure whether you can use a map or not, please do reach out and we will be able to help you. The following websites are good examples of where you can source open access or public domain maps: 
* U.S. Geological Survey (USGS) - All maps are in the public domain. ([http://www.usgs.gov](http://www.usgs.gov/)) 
* PlaniGlobe - All maps are published under a Creative Commons license so please cite “PlaniGlobe, [http://www.planiglobe.com](http://www.planiglobe.com/), CC BY 2.0” in the image credit after the caption. (<http://www.planiglobe.com/?lang=enl>) 
* Natural Earth - All maps are public domain. (<http://www.naturalearthdata.com/about/terms-of-use/>)

Answer

Corrected and the source of the base layer of the map was declared.

Comment
4. We have noticed that you have uploaded Supporting Information files, but you have not included a list of legends. Please add a full list of legends for your Supporting Information files after the references list.

Answer

Supporting information files have been added by mistake. Have uploaded files according to the correct category. No supporting information files (hence the list of legends) are needed.

Additional Editor Comments (if provided):


[Note: HTML markup is below. Please do not edit.]

Reviewers' comments:

Reviewer's Responses to Questions

**Comments to the Author**

1. Does this manuscript meet PLOS Global Public Health’s [publication criteria](https://journals.plos.org/globalpublichealth/s/criteria-for-publication)? Is the manuscript technically sound, and do the data support the conclusions? The manuscript must describe methodologically and ethically rigorous research with conclusions that are appropriately drawn based on the data presented.

Reviewer #1: Partly

2. Has the statistical analysis been performed appropriately and rigorously?

Reviewer #1: I don't know

3. Have the authors made all data underlying the findings in their manuscript fully available (please refer to the Data Availability Statement at the start of the manuscript PDF file)?

The [PLOS Data policy](https://journals.plos.org/globalpublichealth/s/data-availability) requires authors to make all data underlying the findings described in their manuscript fully available without restriction, with rare exception. The data should be provided as part of the manuscript or its supporting information, or deposited to a public repository. For example, in addition to summary statistics, the data points behind means, medians and variance measures should be available. If there are restrictions on publicly sharing data—e.g. participant privacy or use of data from a third party—those must be specified.

Reviewer #1: Yes

Comment

4. Is the manuscript presented in an intelligible fashion and written in standard English?

PLOS Global Public Health does not copyedit accepted manuscripts, so the language in submitted articles must be clear, correct, and unambiguous. Any typographical or grammatical errors should be corrected at revision, so please note any specific errors here.

Reviewer #1: No

Answer

We used an expert in English language to go through the manuscript and used Grammerly software, to correct the issues regarding English, and have edited the new manuscript accordingly.

5. Review Comments to the Author

Please use the space provided to explain your answers to the questions above. You may also include additional comments for the author, including concerns about dual publication, research ethics, or publication ethics. (Please upload your review as an attachment if it exceeds 20,000 characters)

Reviewer #1: Dear Authors:

I have read your manuscript. As you have mentioned the prevalence of GERD is rising all over the world, and not only in the western world. As the first country-wide epidemiologic study is important and will be read and cited is global epidemiological studies and as you are filling the missing data is of importance.

As a general and in detail view of the manuscript there are some points to say:

Comment
• The manuscript is very long and there are many repeated items in the text that could be omitted

Answer

Thank you very much for the comment. Revised manuscript is shortened and repetitions are removed.

Comment
• The main structure is like a research report rather that a medical journal manuscript. You should not tell all of the findings you had found in the study, which were obvious and adds nothing to global knowledge. I think it is better to focus on your best and the most important findings and the items that are different from other parts of the world or alike the other parts that are important to mention.

Answer

Manuscript edited accordingly and most important results are highlighted.

Comment
• I think you have used the term of “reflux” instead of “(acid) regurgitation” interchangeably in the text. The symptom regurgitation differs from the reflux process and reflux disease!

Answer

Understood. The term “regurgitation” is inserted instead of reflux when talking about symptoms.

Comment
• In the sample enrollment process, there is nothing about how you came to the number of 1200 people and how you made your clusters from the country. You had lost some 50 persons due to some reasons that is obvious, but I do not think that the method you enrolled new people is methodologically correct.

Answer

Given below is the methodology of the study in full as written in the PhD thesis it is part of. The manuscript was previously written to keep the length less. But we will add some more details regarding the methodology to be clearer in the revised manuscript with reference for statistical analysis given.

“The total sample size was 1200.

The sample size was calculated using the following formula (Lachenbruch et al., 1991).

n	=	Z_α_^2^	x p (1-p)

d^2^

Z_α_ = 1.96 (Z_α_ value corresponding to a confidence interval of 95% and an α error of 0.05)

d	= 0.05 (level of precision)

n - required sample size

d- degree of precision desired for margin or error set at 0.05

p- expected prevalence

The exact value of the prevalence of GERD in the community is not known in Sri Lanka. As there are no previous estimates of GERD in Sri Lanka, this proportion will be considered as 50% in the calculation to obtain the highest sample size.

Therefore, n =	1.96^2^	x 0.50 x (0.50) = 384.16

0.05^2^

Based on an expected prevalence of 50% with 0.05 precision, at a 95% confidence interval, along with an allowance for design effect cluster sampling of 2 and an anticipated non-response of 5%, the required minimum sample size was rounded off to be 810. Since this study intended to cover each 25 districts of the country the sample size was increased to 1200 to have 40 clusters distributed amongst the 25 districts keeping in mind the probability proportionate to the size (PPS) of the population.

**Sampling method and study procedure**

The 2012 census data was used as the sampling frame which is the most recently available population data from the Sri Lanka Department of Census and Statistics. To use the framework for the probability proportionate to the size (PPS) of the population, the total population of people between 18 to 70 years was taken as 12545123, the cluster number was taken as 40, the cluster interval was calculated as 258432, with a random number of 9.

A group of adults in a Grama Niladhari (GN) division (the smallest administrative unit in Sri Lanka) was defined as a cluster with a cluster size was taken as 30. Each district was allocated several clusters depending on probability proportionate to population (see Table 8.1). Clusters were identified using simple random sampling of the list of the GN divisions of each district (see Table 8.2).

**Table 8.1: Framework for the probability proportionate to the size (PPS) of the population.**

|  | **District** | **Total population** | **Population between 20-70 years** | **Cumulative population** | **Number of clusters** |
| --- | --- | --- | --- | --- | --- |
| 1 | **Ampara** | 648343 | 377984 | 377984 | 2 |
| 2 | **Anuradhapura** | 856399 | 524973 | 902957 | 1 |
| 3 | **Badulla** | 815253 | 499750 | 1402707 | 1 |
| 4 | **Batticoloa** | 525399 | 297376 | 1700083 | 1 |
| 5 | **Colombo** | 2310136 | 1517759 | 3217842 | 4 |
| 6 | **Galle** | 1059069 | 648150 | 3865992 | 2 |
| 7 | **Gampaha** | 2294806 | 1489329 | 5355321 | 4 |
| 8 | **Hambantota** | 596788 | 365831 | 5721152 | 1 |
| 9 | **Jaffna** | 583613 | 350751 | 6071903 | 1 |
| 10 | **Kalutara** | 1217566 | 759761 | 6831664 | 3 |
| 11 | **Kandy** | 1370247 | 835851 | 7667515 | 2 |
| 12 | **Kegalle** | 836659 | 524585 | 8192100 | 1 |
| 13 | **Kilinochchi** | 112980 | 62478 | 8254578 | 1 |
| 14 | **Kurunegala** | 1610955 | 1013291 | 9267869 | 2 |
| 15 | **Mannar** | 99107 | 54013 | 9321882 | 1 |
| 16 | **Matale** | 482406 | 296197 | 9618079 | 1 |
| 17 | **Matara** | 809617 | 484151 | 10102230 | 2 |
| 18 | **Monaragala** | 448210 | 275201 | 10377431 | 1 |
| 19 | **Mullaitivu** | 90842 | 52870 | 10430301 | 1 |
| 20 | **Nuwara Eliya** | 706726 | 421915 | 10852216 | 1 |
| 21 | **Polonnaruwa** | 403782 | 249941 | 11102157 | 1 |
| 22 | **Puttalam** | 760078 | 461367 | 11563524 | 2 |
| 23 | **Ratnapura** | 1082838 | 678939 | 12242463 | 2 |
| 24 | **Trincomalee** | 378255 | 211823 | 12454286 | 1 |
| 25 | **Vavuniya** | 171390 | 90837 | 12545123 | 1 |
| **Total** | | | | **12545123** | **40** |

Random selection was used to identify each cluster's index house using a GN division map. Every third house starting from the right of the index house was approached to make up the cluster. After applying inclusion and exclusion criteria only one member from each family was randomly selected by drawing lots. After obtaining informed written consent he/ she was recruited as a participant.

**Table 8.2: List of GN divisions identified for the study**

| **District** | **GN Division** |
| --- | --- |
| 1. **Ampara** | 1. W/89I - Nawagampura |
|  | 2. 89B - Valathapitty 1 |
| 2. **Anuradhapura** | 3. 358 - Oyamaduwa |
| 3. **Badulla** | 4. 63A - Haputale town |
| 4. **Batticaloa** | 5. 150A - Kirankulam south |
| 5. **Colombo** | 6. 445 - Pahala Hanwella |
|  | 7. 536A - Udyanaya |
|  | 8. Wanathamulla |
|  | 9. Grandpass South |
| 6. **Galle** | 10. 51 - Dodanduwa |
|  | 11. 82A - Hirewatta |
| 7. **Gampaha** | 12. 43B - Kithulwala north |
|  | 13. 239 - Belummahara |
|  | 14. 223 - Medagama 1 |
|  | 15. 36B - Muddaragama |
| 8. **Hambantota** | 16. 032 - Ellagala |
| 9. **Jaffna** | 17. 112 - Tirunelveli southeast |
| 10. **Kalutara** | 18. 754 - Maradana |
|  | 19. 694B - Diganathuduwa |
|  | 20. 756 - Maligahena |
| 11. **Kandy** | 21. 598 - Udurawana |
|  | 22. 603 - Walpaladeniya |
| 12. **Kegalle** | 23. 52A - Beragala |
| 13. **Kilinochchi** | 24. KN/21 - Thiruvaiaru west |
| 14. **Kurunegala** | 25. 841 - Kurunegala town South |
|  | 26. 797 - Bamunugedara 1 |
| 15. **Mannar** | 27. MN74 - Emilnagar |
| 16. **Matale** | 28. 352C - Maligathenna 2 |
| 17. **Matara** | 29. 417H - Weragampita |
|  | 30. 448C - Agarawala |
| 18. **Monaragala** | 31. 134A - Karavila |
| 19. **Mullaitivu** | 32. 61 - Oddusuddan |
| 20. **Nuwara Eliya** | 33. 535E - Sandathenna |
| 21. **Polonnaruwa** | 34. 154 - Samudragama |
| 22. **Puttalam** | 35. 659 - Wadigamangawa |
|  | 36. 656B - Thammennagama 2 |
| 23. **Ratnapura** | 37. 182D - Mudduwa East |
|  | 38. 152B-Weralupe |
| 24. **Trincomalee** | 39. 244E - Villundy |
| 25. **Vavuniya** | 40. 214D - Vairavapuliyankulam |

Additional recruitment when the initial selected participant refused was as follows:

Forty-one subjects who were approached refused or were unable to participate in the study (3.3%). Additional participants (age and sex matched) were recruited from the same cluster to achieve a sample size of 1200.

Same random sampling method was used in recruiting additional participants. Every third house from the point where recruitment stopped was evaluated until age and sex-compatible willing participant/s was recruited. This method ensured the additional participants recruited was from the same cluster and neighborhood and compatible with the participants who refused in age and sex.

Comment
• The term “bloating” as a symptom of GERD disease seems not proper. You may have considered “belching”. If otherwise please give some reference to this symptom in GERD patients.

Answer

Bloating is the feeling that one’s stomach is full or stretched. It is a common symptom in many GI diseases and not specific to GERD. The commonest causes of bloating include functional GI disorders such as IBS, functional dyspepsia, aerophagia, and gastroparesis.

Belching, especially gastric belching, commonly occurs due to gaseous distention of the stomach in conditions such as gastroparesis and aerophagia.

Bloating and belching are two totally different symptoms, even though they can co-exist in some patients.

In patients with GERD symptoms both bloating and belching can occur simultaneously and separately due to two main reasons.

1. A significant proportion of patients with GERD symptoms have functional dyspepsia and not true GERD and bloating is a common symptom in them.
2. Impaired gastric motility or gastroparesis is a well-recognized cause of GERD. Bloating is the most prominent presenting symptom in patients with gastroparesis.

Because of these reasons, we believe that assessing both these symptoms is important in our cohort of patients with GERD symptoms. Previous worldwide studies have also reported both symptoms in patients with GERD (reference 1 and 2).

In addition bloating is a symptom used in the country-validated GERD screening tool (only one available for Sri Lanka) which we used in this study (reference 3).

Due to the lack of gold-standard investigations such as endoscopy and MII-pH used in community studies, we are using screening tools as done in studies worldwide on the prevalence of GERD.

However, according to the reviewer's comments to rectify this ambiguity, we have changed the wording where appropriate from “GERD” to “probable GERD” or “GERD symptoms” in the revised manuscript.

References:

1. Mark Shacker, Andrés R Latorre-Rodríguez, Sumeet K Mittal, Prevalence and severity of abdominal bloating in patients with gastroesophageal reflux disease, Diseases of the Esophagus, 2023;, doad058, <https://doi.org/10.1093/dote/doad058>
2. Sawada, A., Fujiwara, Y., & Sifrim, D. (2020). Belching in Gastroesophageal Reflux Disease: Literature Review. *Journal of clinical medicine*, *9*(10), 3360. <https://doi.org/10.3390/jcm9103360>
3. Amarasiri LD, Pathmeswaran P, De Silva AP, Dassanayake AS, Ranasinha CD, De Silva J. Comparison of a composite symptom score assessing both symptom frequency and severity with a score that assesses frequency alone: a preliminary study to develop a practical symptom score to detect gastro-oesophageal reflux disease in a resource-poor setting. *Eur J Gastroenterol Hepatol*. 2010;22(6):662-668. doi:10.1097/MEG.0B013E32832CFE12

Comment

• Many items in the tables and main text can be shortened to include only the necessary points and facts to be shown.

Answer

We have shortened the manuscript as advised. The content in tables and text are revised to increase the clarity

Comment

• In the discussion section there is a section, we it is said that NSAIDs can induce GERD. They are a well-known etiology for upper GI erosions/ ulcers and also dyspeptic symptoms, but not a main factor in the GERD.

Answer

Most recent systematic reviews and meta-analyses on GERD, done by Eusebi et al in 2016 and Nirwan et al in 2020, included NSAIDs as a risk factor for GERD symptoms (reference 4 and 5). In accordance with this previous meta-analysis, we also included information on NSAIDs in our epidemiological study.

However, as the reviewer correctly mentioned, it is not a main factor for GERD etiology but for gastritis and ulcers, etc., whose symptoms may/ may not overlap with that of a range of GERD symptoms.

In this epidemiological study, we assessed patients with GERD symptoms who were not diagnosed with GERD. Therefore, it was important to inquire about possible risk factors for GERD symptoms including NSAID use. We hope that changing the wording from “GERD” to “probable GERD” or “GERD symptoms” where appropriate with further clarify this matter.

References:

4. Eusebi LH, Ratnakumaran R, Yuan Y, et al. Global prevalence of, and risk factors for, gastro-oesophageal reflux symptoms: A meta-analysis. *Gut*. 2018;67(3):430-440. doi:10.1136/gutjnl-2016-313589
5. Nirwan JS, Hasan SS, Babar ZUD, Conway BR, Ghori MU. Global Prevalence and Risk Factors of Gastro-oesophageal Reflux Disease (GORD): Systematic Review with Meta-analysis. *Scientific Reports 2020 10:1*. 2020;10(1):1-14. doi:10.1038/s41598-020-62795-1

Comment
• For figure 1, I think the box plot type may be a better format concerning the age group chosen in the chart

Answer

We attempted to convert this graph into a box plot according to the reviewer's comments. However, since, three categories of data (male, female, and total) were presented in this graph, when it was converted to a box plot, it was very complex, crowded, and unclear. Therefore, we prefer to keep this figure as a line diagram.

In addition, we like to show the trend of changing prevalence of GERD symptoms, according to age and we believe that a line diagram is best for this.

However, we revised this figure to make it clearer.

Comment

While revising your submission, please upload your figure files to the Preflight Analysis and Conversion Engine (PACE) digital diagnostic tool, <https://pacev2.apexcovantage.com/>. PACE helps ensure that figures meet PLOS requirements. To use PACE, you must first register as a user. Registration is free. Then, login and navigate to the UPLOAD tab, where you will find detailed instructions on how to use the tool. If you encounter any issues or have any questions when using PACE, please email PLOS at [figures@plos.org](mailto:figures@plos.org). Please note that Supporting Information files do not need this step.

Answer

Both figures, uploaded issues sorted using PACE.
